# 普乐沙福联合粒细胞集落刺激因子在淋巴瘤患者中进行自体造血干细胞动员的效果及安全性

**DOI:** 10.3760/cma.j.issn.0253-2727.2023.02.005

**Published:** 2023-02

**Authors:** 濛濛 纪, 一格 沈, 吉昌 龚, 暐 唐, 晓倩 许, 重 郑, 思远 陈, 洋 赫, 欣 郑, 林娣 赵, 维莅 赵, 文 吴

**Affiliations:** 上海血液学研究所，医学基因组学国家重点实验室，国家转化医学中心，上海交通大学医学院附属瑞金医院，上海 200025 Shanghai Institute of Hematology, State Key Laboratory of Medical Genomics, National Research Center for Translational Medicine at Shanghai, Ruijin Hospital Affiliated to Shanghai Jiao Tong University School of Medicine, Shanghai 200025, China

**Keywords:** 普乐沙福, 粒细胞集落刺激因子, 淋巴瘤, 自体造血干细胞动员, Plerixafor, Granulocyte colony stimulating factor, Lymphoma, Autologous hematopoietic stem cell mobilization

## Abstract

**目的:**

分析普乐沙福（Plerixafor）联合粒细胞集落刺激因子（G-CSF）在淋巴瘤患者中进行自体造血干细胞动员的效果及安全性。

**方法:**

收集2019年4月至2021年12月在上海交通大学医学院附属瑞金医院采用普乐沙福联合G-CSF（普乐沙福+G-CSF组）或单用G-CSF（G-CSF组）进行自体造血干细胞动员的淋巴瘤患者，回顾性分析两组之间临床资料、干细胞动员/采集成功率、移植后造血重建和治疗不良反应。

**结果:**

全部184例淋巴瘤患者包括弥漫大B细胞淋巴瘤115例（62.5％）、经典型霍奇金淋巴瘤16例（8.7％）、滤泡性非霍奇金淋巴瘤11例（6.0％）、血管免疫母细胞T细胞淋巴瘤10例（5.4％）、套细胞淋巴瘤6例（3.3％）、间变大细胞淋巴瘤6例（3.3％）、NK/T细胞淋巴瘤6例（3.3％）、伯基特淋巴瘤4例（2.2％）、其他类型B细胞淋巴瘤8例（4.3％）、其他类型T细胞淋巴瘤2例（1.1％）。31例（16.8％）患者曾接受放疗。普乐沙福+G-CSF组84例，G-CSF组100例。普乐沙福+G-CSF组患者年龄较大、复发及三线化疗患者占比较高，其他临床特征基本一致。G-CSF组动员后1 d采集成功率为74.0％，2 d采集成功率为89.0％；普乐沙福+G-CSF组1 d采集成功率为85.7％，2 d采集成功率为97.6％。普乐沙福+G-CSF组的动员成功率明显高于单用G-CSF组（*P*＝0.023）。普乐沙福+G-CSF组、G-CSF组采集CD34^+^细胞中位数分别为3.9（0.7～21.2）×10^6^/kg、3.2（0.6～19.4）×10^6^/kg（*P*＝0.001）。普乐沙福+G-CSF组常见的不良反应为1～2级胃肠道反应（31.2％）、局部皮肤发红（2.4％）。

**结论:**

普乐沙福联合G-CSF用于淋巴瘤患者自体造血干细胞动员/采集成功率、CD34^+^细胞采集量均明显高于单用G-CSF，不良反应少，即使在年龄较大、二线动员、复发或者多次化疗的患者中也具有较高的动员成功率。

## Abstract (en)

**Objective:**

To evaluate the advantages and safety of Plerixafor in combination with granulocyte colony-stimulating factor（G-CSF）in autologous hematopoietic stem cell mobilization of lymphoma.

**Methods:**

Lymphoma patients who received autologous hematopoietic stem cell mobilization with Plerixafor in combination with G-CSF or G-CSF alone were obtained. The clinical data, the success rate of stem cell collection, hematopoietic reconstitution, and treatment-related adverse reactions between the two groups were evaluated retrospectively.

**Results:**

A total of 184 lymphoma patients were included in this analysis, including 115 cases of diffuse large B-cell lymphoma（62.5％）, 16 cases of classical Hodgkin's lymphoma（8.7％）, 11 cases of follicular non-Hodgkin's lymphoma（6.0％）, 10 cases of angioimmunoblastic T-cell lymphoma（5.4％）, 6 cases of mantle cell lymphoma（3.3％）, and 6 cases of anaplastic large cell lymphoma（3.3％）, 6 cases of NK/T-cell lymphoma（3.3％）, 4 cases of Burkitt's lymphoma（2.2％）, 8 cases of other types of B-cell lymphoma（4.3％）, and 2 cases of other types of T-cell lymphoma（1.1％）; 31 patients had received radiotherapy（16.8％）. The patients in the two groups were recruited with Plerixafor in combination with G-CSF or G-CSF alone. The baseline clinical characteristics of the two groups were basically similar. The patients in the Plerixafor in combination with the G-CSF mobilization group were older, and the number of recurrences and third-line chemotherapy was higher. 100 patients were mobilized with G-CSF alone. The success rate of the collection was 74.0％ for one day and 89.0％ for two days. 84 patients in the group of Plerixafor combined with G-CSF were recruited successfully with 85.7％ for one day and 97.6％ for two days. The success rate of mobilization in the group of Plerixafor combined with G-CSF was substantially higher than that in the group of G-CSF alone（*P*＝0.023）. The median number of CD34^+^ cells obtained in the mobilization group of Plerixafor combined with G-CSF was 3.9×10^6^/kg. The median number of CD34^+^ cells obtained in the G-CSF Mobilization group alone was 3.2×10^6^/kg. The number of CD34^+^ cells collected by Plerixafor combined with G-CSF was considerably higher than that in G-CSF alone（*P*＝0.001）. The prevalent adverse reactions in the group of Plerixafor combined with G-CSF were grade 1–2 gastrointestinal reactions（31.2％）and local skin redness（2.4％）.

**Conclusion:**

The success rate of autologous hematopoietic stem cell mobilization in lymphoma patients treated with Plerixafor combined with G-CSF is significantly high. The success rate of collection and the absolute count of CD34^+^ stem cells were substantially higher than those in the group treated with G-CSF alone. Even in older patients, second-line collection, recurrence, or multiple chemotherapies, the combined mobilization method also has a high success rate of mobilization.

淋巴瘤是血液系统最常见的恶性肿瘤之一，其发病率及死亡率表现为逐年上升趋势，2020年美国癌症统计报告，仅非霍奇金淋巴瘤发病率及其死亡率已上升至所有癌症的第7位[1]。目前治疗方案包括化学治疗、局部放射治疗、自体造血干细胞移植（auto-HSCT）及细胞免疫治疗等。大剂量化疗联合auto-HSCT能够明显延长患者的生存时间，改善预后，被推荐为复发难治性和（或）高侵袭性淋巴瘤的巩固治疗方案。自体造血干细胞动员效率是auto-HSCT成功的关键，采集足够数量的造血干细胞才能保证移植的顺利进行、移植后造血快速重建及疾病的较好转归[2]。

自体造血干细胞动员方法包括粒细胞集落刺激因子（G-CSF）单药或联合普乐沙福或联合大剂量化疗。单用G-CSF的患者采集的细胞数有限，采集的失败率较高。G-CSF联合大剂量化疗的动员方案存在采集时间难以预测、住院时间长、化疗相关不良反应多、治疗风险及费用增加等缺点[3]。普乐沙福是趋化因子受体4（CXCR4）的抑制剂，一旦进入骨髓，与干细胞表面CXCR4结合后使其失锚定直接进入血液[4]。普乐沙福联合G-CSF可以显著提高CD34^+^细胞采集量，降低动员失败率，缩短采集天数[5]。为了进一步研究普乐沙福在临床中实际应用的效果，我们对普乐沙福联合G-CSF、单用G-CSF两种方案进行干细胞动员采集的淋巴瘤患者进行比较分析。

## 病例与方法

一、病例

本回顾性研究纳入2019年4月至2021年12月期间在上海交通大学附属瑞金医院采用普乐沙福联合G-CSF（普乐沙福+G-CSF组）、单用G-CSF（G-CSF组）进行自体造血干细胞动员的184例淋巴瘤患者。根据患者的组织病理、骨髓形态学、免疫学、细胞遗传学、分子生物学、增强电子计算机断层扫描（CT）、磁共振成像（MRI）和正电子发射断层成像（PET-CT）明确诊断。本研究获得上海交通大学附属瑞金医院伦理委员会的批准。

二、治疗方案

参照国内外淋巴瘤治疗指南，依据患者疾病分类、分期及分子生物学等特点，采用（R）CHOP、（R）DA-EPOCH、Hyper-CVAD等方案为基础的联合化疗方案治疗，视患者具体病情决定是否联合受累野放疗，视患者疾病缓解情况，3～6疗程后行外周血自体造血干细胞动员采集。

三、动员及采集方案

普乐沙福应用指证：①患者采用普乐沙福联合G-CSF或单用G-CSF行干细胞动员，G-CSF动员过程中监测CD34^+^细胞数，第4天预计CD34^+^细胞数不能达标者，予以抢先联合普乐沙福动员；②预计采集成功率低、既往使用G-CSF动员失败的患者再次动员时优先选择联合普乐沙福动员；③对采集成功率、就诊满意度有较高要求的患者优先选择联合动员方案[6]。

给药方案：①G-CSF 10 µg·kg^−1^·d^−1^第1～4天皮下注射；②普乐沙福1.2 ml，第4天22:00皮下注射；③第5天9:00开始采集，提前1 h皮下注射G-CSF，采集患者外周血标本，行CD34^+^细胞及单个核细胞（MNC）计数。

自体造血干细胞采集目标为CD34^+^细胞≥2.0×10^6^/kg。用流式细胞术检测CD34^+^细胞计数及其百分比。

四、auto-HSCT预处理方案及植入标准

依据患者病理类型，分别选用BEAM（卡莫司汀+依托泊苷+阿糖胞苷+美法仑）、BEAC（卡莫司汀+依托泊苷+阿糖胞苷+环磷酰胺）、Bu/Cy+E（白消安+环磷酰胺+依托泊苷）、卡莫司汀+塞替派方案进行预处理，自WBC<2.0×10^9^/L起予以G-CSF，至中性粒细胞植入停药。植入标准：中性粒细胞计数（ANC）≥0.5×10^9^/L连续3 d为中性粒细胞植入；PLT≥20.0×10^9^/L连续7 d且脱离血小板输注为血小板植入。

五、定义、疗效疗效评估标准及安全性评估

淋巴瘤疗效评价标准参照参照Lugano淋巴瘤疗效评估标准，不良反应采用CTCAE5.0版分级标准。

六、统计学处理

采用SPSS 22.0软件进行描述性统计分析。组间临床差异和移植后造血重建时间采用卡方检验和*t*检验进行比较。Kaplan-Meier法分析生存情况及造血恢复差异。以*P*<0.05为差异有统计学意义。

## 结果

一、基线临床特征

共纳入184例患者，其中G-CSF组100例，普乐沙福+G-CSF组84例。G-CSF组100例患者中，T细胞淋巴瘤13例（13.0％），B细胞淋巴瘤73例（73.0％），霍奇金淋巴瘤14例（14.0％）；Ann Arbor Ⅰ/Ⅱ期35例（35.0％），Ann Arbor Ⅲ/Ⅳ期65例（65.0％）；乳酸脱氢酶（LDH）升高（>192 U/L）60例（60.0％），有B症状10例（10.0％），复发患者10例（10.0％），5例（5.0％）患者动员前已接受三线化疗，14例（14.0％）患者动员前接受放疗。普乐沙福+G-CSF组84例患者中，T细胞淋巴瘤11例（13.1％），B细胞淋巴瘤71例（84.5％），霍奇金淋巴瘤2例（2.4％）；Ann Arbor Ⅰ/Ⅱ期21例（25.0％），Ann Arbor Ⅲ/Ⅳ期63例（75.0％），LDH升高55例（65.5％），有B症状14例（16.7％），复发患者19例（22.6％），14例（16.7％）患者动员前已接受三线化疗，17例（20.0％）患者动员前接受放疗。两组患者基线临床特征基本一致，普乐沙福+G-CSF组患者年龄较大（*P*＝0.024），复发及三线化疗患者占比较高（*P*＝0.019，*P*＝0.010）。患者基线临床资料详见表1。

**表1:** 普乐沙福+G-CSF、单用G-CSF两种自体造血干细胞动员方案淋巴瘤患者临床特征比较

指标	G-CSF（100例）	普乐沙福+G-CSF（84例）	统计量	*P*值
年龄［岁，*M*（范围）］	43（15~68）	53（23~68）	5.055（*t*值）	0.024
T-NHL［例（％）］	13（13.0）	11（13.1）	<0.001（*χ*^2^值）	0.985
B-NHL［例（％）］	73（73.0）	71（84.5）	3.563（*χ*^2^值）	0.059
Ann-Arbor分期［例（％）］			2.156（*χ*^2^值）	0.142
Ⅰ+Ⅱ	35（35.0）	21（25.0）		
Ⅲ+Ⅳ	65（65.0）	63（75.0）		
LDH>192 U/L［例（％）］	60（60.0）	55（65.5）	0.584（*χ*^2^值）	0.445
有B症状［例（％）］	10（10.0）	14（16.7）	1.789（*χ*^2^值）	0.181
复发［例（％）］	10（10.0）	19（22.6）	5.475（*χ*^2^值）	0.019
动员前三线治疗［例（％）］	5（5.0）	14（16.7）	6.710（*χ*^2^值）	0.010
动员前放疗［例（％）］	14（14.0）	17（20.0）	1.268（*χ*^2^值）	0.260

**注** T-NHL：T细胞来源非霍奇金淋巴瘤；B-NHL：B细胞来源非霍奇金淋巴瘤；LDH：乳酸脱氢酶；G-CSF：粒细胞集落刺激因子

二、自体造血干细胞采集结果

两种不同动员方案均以外周血CD34^+^细胞≥15/µl为采集成功判定标准[6]。G-CSF组100例，动员后1天采集成功率为74.0％，2 d采集成功率为89.0％，中位CD34^+^细胞采集量为3.2（0.6～19.4）×10^6^/kg。普乐沙福+G-CSF组84例，1 d采集成功率85.7％，2 d采集成功率97.6％，中位CD34^+^细胞采集量为3.9（0.7～21.2）×10^6^/kg。普乐沙福+G-CSF组的动员成功率明显高于G-CSF组（*P*＝0.023），CD34^+^细胞采集量也显著高于G-CSF组（*P*＝0.001）。详见表2。

**表2:** 普乐沙福+G-CSF组、单用G-CSF组淋巴瘤患者自体造血干细胞动员结果比较

指标	G-CSF组（100例）	普乐沙福+G-CSF（84例）	统计量	*P*值
采集成功［例（％）］	89（89.0）	82（97.6）	5.165（*χ*^2^值）	0.023
第1天采集成功［例（％）］	74（74.0）	72（85.7）	1.813（*χ*^2^值）	0.178
第2天采集成功［例（％）］	15（15.0）	10（11.9）	0.305（*χ*^2^值）	0.584
CD34^+^细胞总采集量［×10^6^/L，*M*（范围）］	3.2（0.6~19.4）	3.9（0.7~21.2）	3.393（*t*值）	0.001
CD34^+^细胞第1次采集量［×10^6^/L，*M*（范围）］	3.1（0.6~19.4）	3.9（0.7~21.2）	2.584（*t*值）	0.011
CD34^+^细胞第2次采集量［×10^6^/L，*M*（范围）］	1.0（0.6~19.4）	0.8（0.1~3.1）	2.071（*t*值）	0.047

三、移植情况及造血重建

G-CSF组100例患者中62例行auto-HSCT，中位回输CD34^+^细胞数为3.2（1.6～19.4）×10^6^/kg，中性粒细胞植入时间为10（8～17）d，血小板植入时间为12（9～27）d。普乐沙福+G-CSF组84例患者中60例行auto-HSCT，中位回输CD34^+^细胞数为4.0（2.0～11.6）×10^6^/kg，中性粒细胞植入时间为10（8～16）d，血小板植入时间为12（9～22）d。两组的造血重建时间没有明显差异。两组患者移植情况及造血重建情况详见表3。

**表3:** 普乐沙福+G-CSF、G-CSF两种自体造血干细胞动员方案淋巴瘤患者移植情况

指标	G-CSF组（62例）	普乐沙福+G-CSF组（60例）	统计量	*P*值
性别［例（％）］			2.090（*χ*^2^值）	0.148
男	42（67.7）	33（55.0）		
女	20（32.3）	27（45.0）		
复发［例（％）］	6（9.6）	13（21.7）	3.333（*χ*^2^值）	0.037
移植前疾病状态［例（％）］			0.274（*χ*^2^值）	0.601
完全缓解	55（88.7）	54（90.0）		
部分缓解	7（11.3）	6（10.0）		
移植预处理方案［例（％）］			7.150（*χ*^2^值）	0.128
BEAM	49（79.0）	44（73.4）		
Bu/Cy-E	6（9.6）	13（21.6）		
BEAC	3（4.8）	0（0）		
BEAC+克拉屈滨	1（1.6）	2（3.3）		
卡莫司汀+塞替派	3（4.8）	1（1.7）		
回输CD34^+^细胞［×10^6^/L，*M*（范围）］	3.2（1.3~19.4）	4.0（2.0~11.6）	2.469（*t*值）	0.015
粒细胞植入时间［d，*M*（范围）］	10（8~17）	10（8~16）	0.979（*t*值）	0.330
血小板植入时间［d，*M*（范围）］	12（9~27）	12（9~22）	0.650（*t*值）	0.517

**注** BEAM方案：卡莫司汀+依托泊苷+阿糖胞苷+美法仑；Bu/Cy-E方案：白消安+环磷酰胺+依托泊苷；BEAC方案：卡莫司汀+依托泊苷+阿糖胞苷+环磷酰胺

四、采集失败的高危因素分析

干细胞动员失败的13例患者中，2例（15.4％）分期较晚、LDH升高且接受二线及以上治疗，2例（15.4％）分期较晚且接受二线以上治疗，2例（15.4％）LDH升高且接受二线及以上治疗，1例（6.67％）分期较晚且LDH升高，3例（20.0％）患者LDH升高，2例（15.4％）患者分期较晚，1例患者接受二线及以上治疗。这些相关的因素可能是导致动员不佳、采集失败的原因。我们对干细胞动员/采集失败组和干细胞动员/采集成功组的基线临床特征进行了对比分析，未发现明显的组间差异。干细胞动员/采集失败组与干细胞动员/采集成功组的临床特征比较见表4。

**表4:** 自体造血干细胞采集失败与采集成功淋巴瘤患者临床特征比较

指标	采集失败（13例）	采集成功（171例）	统计量	*P*值
年龄［岁，*M*（范围）］	46（30~60）	48（15~68）	0.147（*t*值）	0.883
Ann Arbor分期［例（％）］			1.633（*χ*^2^值）	0.201
Ⅰ/Ⅱ	6（46.2）	50（29.2）		
Ⅲ/Ⅳ	7（53.8）	121（80.8）		
LDH升高［例（％）］	7（53.8）	108（63.2）	0.447（*χ*^2^值）	0.504
有B症状［例（％）］	1（7.7）	23（13.4）	0.353（*χ*^2^值）	0.552
复发［例（％）］	2（15.4）	26（15.2）	<0.001（*χ*^2^值）	0.986
动员前治疗≥2线［例（％）］	7（53.8）	127（74.0）	2.546（*χ*^2^值）	0.111
动员前放疗［例（％）］	1（7.7）	29（17.0）	0.760（*χ*^2^值）	0.383

五、不良反应

普乐沙福+G-CSF组常见的不良反应为1～2级胃肠道反应（31.2％），表现为注射2～3 h后出现腹泻，不伴腹痛，予以对症处理，次日采集结束后症状消失。其余不良表现包括局部皮肤发红（2.4％）等，均自行缓解。

## 讨论

auto-HSCT目前已成为淋巴瘤重要的巩固治疗手段，动员、采集到足够数量的CD34^+^细胞是auto-HSCT成功的关键步骤之一。

准确把握采集最佳时机是能够获得足够数量造血干细胞的核心环节。目前研究显示，外周血CD34^+^细胞数与采集物中干细胞绝对值计数正相关，不仅能够帮助确定采集时机，同时可以预测采集量及采集次数，通过流式细胞术可以检测外周血CD34^+^细胞数。外周血CD34^+^细胞数≥20个/µl被认为是采集成功率极高的标志，CD34^+^细胞数绝对值<5个/µl则不推荐干细胞采集[6]。CD34^+^细胞数15～20/µl时，第1天采集1.3×10^6^/kg干细胞，当CD34^+^细胞10～15/µl时，第1天采集干细胞数<1.0×10^6^/kg，而第1天采集<1.0×10^6^/kg被认为是动员失败的标志[7]。本研究中，普乐沙福+G-CSF组患者单采第1天测定CD34^+^细胞中位数为42.7个/µl，确定CD34^+^细胞>15/µl（96.4％）为合适采集时机。关于使用普乐沙福促进自体造血干细胞动员，英国共识推荐当单采第1天CD34^+^细胞<15/µl时使用普乐沙福；当CD34^+^细胞15～20/µl时，依据患者后续疾病治疗需求决定是否加用普乐沙福[8]。本研究中，普乐沙福+G-CSF组患者年龄较大、复发及三线化疗患者占比较高，采集成功率明显高于G-CSF组（97.6％对89.0％，*P*＝0.023）。普乐沙福作为一种新型动员剂，在临床使用过程中，即使是对于有动员失败高危因素的患者，仍表现出了稳定、高质量动员的特征。Haverkos等[9]也得出了同样的结论。Milonea等[10]研究也表明，按最高费用获益比予以普乐沙福的动员成功率及采集成功率均可以达到97.2％。

传统干细胞动员方法包含单独使用细胞因子或联合化疗，动员后CD34^+^细胞数量不足、最佳采集时间难以确定、采集次数增多等因素是采集过程中面临的短板。Sarici等[11]采用挽救性化疗方案（DHAP、ESHAP、ICE）/环磷酰胺联合G-CSF或单用G-CSF为动员方案，采集成功率为90.2％～97.8％，采集次数1～5次。本研究中，普乐沙福+G-CSF组不仅具有较高的采集成功率（97.6％），同时中位采集天数为1（1～2）d，实现稳态动员、可减轻患者的不适并节省医疗资源。

本研究中，普乐沙福+G-CSF组总体动员失败率仅为2.4％，采集CD34^+^细胞总数3.9×10^6^/kg，高于G-CSF组（*P*＝0.001）。auto-HSCT过程中，两组中性粒细胞植入时间均为10 d，血小板植入时间均为12 d。同样也有研究表明，G-CSF联合化疗与单用G-CSF动员后行auto-HSCT的患者中，中性粒细胞及血小板植入时间无显著差异[11]。研究显示，使用普乐沙福联合G-CSF或单用G-CSF行干细胞动员的淋巴瘤患者，auto-HSCT后5年OS及PFS差异无统计学意义[12]。本研究随访时间尚不足以分析其OS、PFS等预后情况。

目前较公认动员不佳的高危因素包括治疗相关（过多的化疗及化疗中使用的部分药物、放疗）、患者相关（疾病复发、老年、糖尿病）及骨髓（骨髓受累）相关因素[13]–[14]，同时淋巴瘤的疾病类型可能也是动员不佳的高危因素[15]。本研究干细胞动员失败的13例患者中，2例（15.4％）分期较晚、LDH升高且接受二线及以上治疗，2例（15.4％）分期较晚且接受二线以上治疗，2例（15.4％）LDH升高且接受二线及以上治疗，1例（6.67％）分期较晚且LDH升高，3例（20.0％）患者LDH升高，2例（15.4％）患者分期较晚，1例患者接受二线及以上治疗，这些相关的因素可能是导致动员不佳、采集失败的原因。此外，包括高龄（年龄>65岁）、罹患糖尿病或者淋巴瘤侵犯骨髓、多周期化疗（≥10个周期）、既往化疗中曾使用来那度胺、氟达拉滨、苯达莫司汀、铂类药物、烷化剂等，也会影响造血干细胞动员成功率，增加二次采集的风险[8]_。_

本研究表明，普乐沙福能够提高淋巴瘤患者自体干细胞采集的成功率及CD34^+^细胞量，减少采集次数，但鉴于其高昂的价格，我们仍建议采用按需应用方案，主要用于①需要较高CD34^+^细胞采集量的患者；②G-CSF单用或联合化疗方案动员过程中实时监测外周血CD34^+^细胞计数预计动员失败的患者；③具有动员失败高危因素且需减少采集天数的患者。

## References

[1] Siegel RL, Miller KD, Jemal A (2020). Cancer statistics, 2020[J]. CA Cancer J Clin.

[2] Klaus J, Herrmann D, Breitkreutz I (2007). Effect of CD34 cell dose on hematopoietic reconstitution and outcome in 508 patients with multiple myeloma undergoing autologous peripheral blood stem cell transplantation[J]. Eur J Haematol.

[3] 中华医学会血液学分会浆细胞疾病学组, 中国医师协会多发性骨髓瘤专业委员会 (2021). 中国多发性骨髓瘤自体造血干细胞移植指南(2021年版)[J]. 中华血液学杂志.

[4] Duong HK, Savani BN, Copelan E (2014). Peripheral blood progenitor cell mobilization for autologous and allogeneic hematopoietic cell transplantation: guidelines from the American Society for Blood and Marrow Transplantation[J]. Biol Blood Marrow Transplant.

[5] Zhu J, Huang H, Chen H (2018). Plerixafor and granulocyte-colony-stimulating factor for mobilization of hematopoietic stem cells for autologous transplantation in Chinese patients with non-Hodgkin's lymphoma: a randomized Phase 3 study[J]. Transfusion.

[6] 中华医学会血液学分会, 中国临床肿瘤学会(CSCO)抗淋巴瘤联盟 (2020). 淋巴瘤自体造血干细胞动员和采集中国专家共识(2020年版)[J]. 中华血液学杂志.

[7] Lemoli RM (2012). New strategies for stem cell mobilization[J]. Mediterr J Hematol Infect Dis.

[8] Douglas KW, Gilleece M, Hayden P (2018). UK consensus statement on the use of plerixafor to facilitate autologous peripheral blood stem cell collection to support high-dose chemoradiotherapy for patients with malignancy[J]. J Clin Apher.

[9] Haverkos BM, Huang Y, Elder P (2017). A single center's experience using four different front line mobilization strategies in lymphoma patients planned to undergo autologous hematopoietic cell transplantation[J]. Bone Marrow Transplant.

[10] Milonea G, Martinob M, Leottaa S (2018). Cost-effectiveness of on-demand plerixafor added to chemotherapy and granulocyte-colony stimulating factor for peripheral blood stem cell mobilization in multiple myeloma[J]. Leuk Lymphoma.

[11] Sarici A, Erkurt MA, Kuku I (2021). Selection of the mobilization regimen in lymphoma patients: A retrospective cohort study[J]. Transfus Apher Sci.

[12] Micallef IN, Stiff PJ, Nademanee AP (2018). Plerixafor plus granulocyte colony-stimulating factor for patients with non-Hodgkin lymphoma and multiple myeloma: long-term follow-up report[J]. Biol Blood Marrow Transplant.

[13] Giralt S, Costa L, Schriber J (2014). Optimizing autologous stem cell mobilization strategies to improve patient outcomes: consensus guidelines and recommendations[J]. Biol Blood Marrow Transplant.

[14] 郭 秋霞, 王 吉刚 (2022). 淋巴瘤患者外周血造血干细胞动员采集过程中相关因素对首次采集结果的影响[J]. 中国组织工程研究.

[15] 苏 永锋, 王 艺志, 宁 红梅 (2021). 淋巴瘤与多发性骨髓瘤患者自体外周血造血干细胞动员影响因素分析[J]. 中国实验血液学杂志.

